# Molecular determinants of WNT9b responsiveness in nephron progenitor cells

**DOI:** 10.1371/journal.pone.0215139

**Published:** 2019-04-12

**Authors:** Kyle K. Dickinson, Leah C. Hammond, Courtney M. Karner, Nicholas D. Hastie, Thomas J. Carroll, Paul Goodyer

**Affiliations:** 1 Department of Experimental Medicine, McGill University, Montreal, Quebec, Canada; 2 Department of Human Genetics, McGill University, Montreal, Quebec, Canada; 3 Department of Orthopaedic Surgery and Cell Biology, Duke University School of Medicine, Durham, North Carolina, United States of America; 4 Medical Research Council Human Genetics Unit, University of Edinburgh, Edinburgh, Scotland; 5 Department of Molecular Biology, University of Texas Southwestern, Dallas, Texas, United States of America; 6 Department of Paediatrics, McGill University Health Centre, Montreal, Quebec, Canada; University of Alabama at Birmingham, UNITED STATES

## Abstract

Primed nephron progenitor cells (NPCs) appear in metanephric mesenchyme by E11.5 and differentiate in response to the inductive WNT9b signal from the ureteric bud. However, the NPC WNT-receptor complex is unknown. We obtained M15 cells from E10.5 mesonephric mesenchyme and systematically analyzed components required for canonical WNT9b-responsiveness. When M15 cells were transfected with a β-catenin luciferase reporter plasmid, exposure to recombinant WNT9b resulted in minimal luciferase activity. We then analyzed mRNA-expression of WNT-pathway components and identified *Fzd1-6* and *Lrp6* transcripts but not *Rspo1*. When M15 cells were treated with recombinant RSPO1 the response to transfected WNT9b was augmented 4.8-fold. Co-transfection of M15 cells with *Fzd5* (but no other *Fzd* family member) further increased the WNT9b signal to 16.8-fold and siRNA knockdown of *Fzd5* reduced the signal by 52%. Knockdown of *Lrp6* resulted in 60% WNT9b signal reduction. We confirmed *Fzd5*, *Lrp6* and *Rspo1* mRNA expression in CITED1(+) NPCs from E15.5 embryonic mouse kidney. Thus, while many WNT signaling-pathway components are present by E10.5, optimum responsiveness of E11.5 cap mesenchyme requires that NPCs acquire RSPO1, FZD5 and LRP6.

## Introduction

The mammalian kidneys are derived from progenitor cells in the embryonic intermediate mesoderm, expressing the transcription factor, OSR1. Fate mapping studies of the embryonic kidney reveal that cells labeled by the *Osr1* promoter at embryonic day E7.5 give rise to all elements of the maturing kidney [1] and *Osr1* knockout mice are anephric [2, 3]. Around E8.5-E9, a subset of OSR1-positive kidney progenitor cells are transformed into polarized epithelia, forming the paired nephric duct structures that elongate down the embryo [4]. Concurrently, another subset of cells upregulate Wilms’ tumor 1 (WT1) while retaining a mesenchymal phenotype. [5, 6]. The columns of WT1(+) cells flanking each nephric duct are committed to the nephron progenitor cell (NPC) fate; interestingly, *Wt1* knockout mice fail to develop functional kidneys [7]. Development of the metanephric kidney begins in earnest when ureteric buds emerge from each nephric duct (E10.5), begins to arborize as it grows into the adjacent column of metanephric mesenchyme and induces local NPCs to begin nephrogenesis.

In the 1950s, Grobstein demonstrated that the metanephric mesenchyme can generate renal tubular structures when co-cultured with inductive tissues that mimic the ureteric bud signal [8]. This fundamental observation showed that the proper signal from the ureteric bud could trigger differentiation in the committed NPCs from the metanephric mesenchyme. Key observations by Herzlinger [9] and Carroll [10, 11] established the canonical WNT9b/β-catenin signaling pathway as the central mechanism by which the ureteric bud initiates nephrogenesis. Secretion of WNT9b by the ureteric bud is required for the early inductive events in the developing kidney. Transgenic mice with a beta-catenin reporter display intense canonical WNT-signaling activity in the cap mesenchyme [12, 13].

It is uncertain when NPCs become competent to respond to the inductive WNT signal, however, WT1 expression is a crucial element in this process. Biallelic mutations of *WT1* in humans result in the formation of nephrogenic rests, clonal developmentally arrested cells which lack canonical WNT-signalling activity and are unresponsive to inductive signals from the ureteric bud [14]. We discovered that this is accomplished by WT1 suppression of EZH2, de-repressing epigenetically silenced genes of the differentiation cascade [15]. Prior to arrival of the ureteric bud (E10.5-E11), maturing WT1(+) NPCs express a panel of genes, including retinoic acid receptor-alpha (*Rara*), cadherin 11 (*Cdh11*) and CD24 [13, 16]. However, the stage at which they are fully competent to respond to the WNT9b signal is unknown. Furthermore, the molecular basis for WNT9b responsiveness in NPCs is unknown.

The canonical WNT signaling pathway is full of redundancies. Here we take a systematic approach to identifying the crucial components of the WNT9b signaling pathway in embryonic mouse kidney.

## Materials and methods

### Cell culture

M15 cells are WT1-expressing cells isolated from E10.5 mouse mesonephric mesenchyme expressing the large T protein of polyoma virus under control of the early viral enhancer. The M15 cell line was establish following the protocol described by Larsson et al (1995) and donated by the Hastie lab (Edinburgh, Scotland) [17]. Cells growing in monolayer attached to plastic culture vessels in the presence of DMEM culture medium with 10% Fetal Bovine Serum and 1% Penicillin/ Streptomycin.

### Luciferase reporter transfections and dual luciferase assay

Transient transfections were performed using a canonical WNT-signalling reporter plasmid, Super 8X TOPFlash (TOPFlash). M50 Super 8x TOPFlash was a gift from Randall Moon (Addgene plasmid # 12456; http://n2t.net/addgene:12456; RRID:Addgene_12456) [18]. The Renilla luciferase expression vector pRL-SV40 (Promega, Madison, WI, USA) was used to normalize for transfection efficiency. Transfections for each condition were performed in triplicate and repeated three times on different days. The following frizzled plasmids were gifts from Chris Garcia & Jeremy Nathans: pRK5-mFzd1-1D4, pRK5-mFzd2-1D4, pRK5-mFzd3-1D4, pRK5-mFzd4-1D4, pRK5-mFzd5-1D4, pRK5-mFzd6-1D4, pRK5-mFzd7-1D4, pRK5-mFzd8-1D4, pRK5-mFzd9-1D4, pRK5-mFzd10-1D4 and pRK5-Wnt9b [19] (Addgene, Cambridge, MA, USA). *Lrp5* (Clone ID: 3154246) and *Lrp6* (Clone ID: 6409058) plasmids were purchased from Dharmachon (Lafayette, CO, USA).

One day prior to transfection, 20,000 M15 cells were seeded in 24-well plates and transfected at 80% confluency using Lipofectamine 2000 Transfection Reagent according to the manufacturer’s instructions (Thermo Fisher Scientific, Waltham, MA, USA). Plasmids were transfected in the following amounts: *Fzd* (50 ng), TOPFlash (44 ng), *Lrp* (5 ng), *Wnt* (50 ng), Renilla (1 ng). Recombinant WNT9b (3669-WN/CF, R&D Systems, Minneapolis, MN, USA) was added at a concentration of 50 ng/mL to transfection media at the time of transfection in corresponding conditions. In R-spondin conditions, either 200 ng/mL of recombinant mouse RSPO1 (3474-RS–R&D Systems, Minneapolis, MN, USA) or 200 ng/mL of recombinant mouse RSPO3 (4120-RS/CF–R&D Systems, Minneapolis, MN, USA) was added to each well 24 hours post transfection. Firefly and renilla luciferase reporter activities were measured after 48h using the Dual Luciferase Assay System reagents and quantified in a GLOMAX 96 microplate luminometer (Promega, Madison, WI, USA). The reporter activity was expressed as a Firefly luciferase/ Renilla luciferase ratio.

The same procedure as described above was followed to monitor luciferase activity. For siRNA experiments, cells were transfected with Silencer pre-designed siRNA targeting mouse *Fzd1* (siRNA ID: 75730), *Fzd2* (siRNA ID: 57265), *Fzd5* (siRNA ID: 14367) and *Lrp6* (siRNA ID: 62715) (Ambion, Carlsbad, CA, USA) using Lipofectamine 2000 transfection reagent (Thermo Fisher Scientific, Waltham, MA, USA) according to manufacturer instructions.

### RNA isolation and real-time PCR analysis

RNA was isolated using the QIAGEN RNeasy kit according to the manufacturer’s instructions (QIAGEN, Toronto, ON, Canada). RT-PCR was performed using the iScript cDNA synthesis kit (Bio-Rad, Mississauga, ON, Canada). Quantitative real-time PCR was performed using the SsoFast EvaGreen Supermix with Low ROX (Bio-Rad, Mississauga, ON, Canada) and specific primer sets in a LightCycler 480 II (Roche Applied Science, Laval, QC, Canada).

### Immunoblotting

Protein content was quantified in cellular extracts using the BCA assay (Pierce, Rockford, IL, USA). Twenty-five micrograms of protein extract were loaded onto SDS-PAGE gel and subjected to electrophoresis following standard immunoblotting techniques. The following primary antibodies and titres were used: anti-WT1 (antibody C19: sc-192, 1/200, Santa Cruz Biotechnology, Santa Cruz, CA, USA), anti-Actin (A5441, 1/10000, Sigma-Aldrich, Oakville, ON, Canada). Immunoreactive bands were detected using species-specific horseradish peroxidase-conjugated secondary antibodies (1/2000, Cell Signaling, Danvers, MA, USA) and visualized and analyzed using the GE Healthcare ECL Plus Western Blotting Detection Reagents and the BioRad Imager Scanner and software (GE Healthcare, Mississauga, ON, Canada).

### In situ hybridization

In situ hybridization of E11.5 embryos was performed according to the protocol listed on the GUDMAP website: https://www.gudmap.org/chaise/recordset/#2/Protocol:Protocol@sort(RID). cDNAs were purchased from ThermoFischer/Open Biosystems. For each gene, we include the clone ID, the restriction enzyme used to linearize the plasmid and the polymerase used to synthesize the antisense probe. *Fzd1* (Clone ID: 5697795) SalI/T3, *Fzd2* (Clone ID: 6411627) SalI/T3, *Fzd3* (Clone ID: 30084926) EcoRI/T3, *Fzd4* (Clone ID: 4238940) SalI/T7, *Fzd5* (Clone ID: UI-M-CGOP-BRL-B-03-0-UI) EcoRI/T3, *Fzd6* (Clone ID: 3983985) SalI/T7, *Fzd7* (Clone ID: 6844727) SalI/T3, *Fzd8* (Clone ID: 3992722) SalI/T7, *Fzd9* (Clone ID: UI-M-CGOP-BGI-E-03-0-UI), *Fzd10* (Clone ID: 556296) Pst1/T7.

### Mice

All animal experiments followed the guidelines provided by the Canadian Council of Animal Care and were approved by the McGill University Facility Animal Care Committee (FACC), including an analysis of the 3Rs of animal use in research. Cited1-Cre mice were donated from Dr. Mark de Caestecker [20]. B6.Cg-Gt(ROSA)26Sortm14(CAG-tdTomato)Hze/J (Tom^flox/flox^)mice were bought from Jackson Laboratories. All animals were housed at the Research Institute of the McGill University Health Centre animal facility and monitored daily by animal care staff. Support staff followed McGill University Standard Operating Procedure #508 for rodent husbandry guidelines (https://mcgill.ca/research/files/research/508_-_rodent_husbandry_-_march_2016_1.pdf). Cited1-Cre males were crossed with homozygous Tom^flox/flox^ females to generate double transgenic embryos. All genotypes generated from this cross were viable and healthy. For immunofluorescence experiments, at 17 dpc, 0.1 mg/g body weight of Tamoxifen (Sigma) was administered to pregnant females via intraperitoneal injection in their home cage [21]. No adverse events were observed in the pregnant female or embryos at this dose of tamoxifen administration. Females were sacrificed 24 hours later, and embryos were harvested. For ddPCR experiments on Cited1/Tom cells, pairs of embryonic kidneys were plated in a single well of a 6-well plate after digestion in a collagenase B digestion solution at 37˚C for 1 hour. These cells were subsequently treated with 2.5 μg/mL of 4-hydroxytamoxifen added to culture media. Digested embryonic kidneys from one pregnancy were pooled and cells were grown at 37˚C in tissue culture flasks in NPC growth media [22].

### Tissue preparation and confocal microscopy

Embryonic mouse kidneys (E18) from Cited1/Tom mice were fixed overnight in 4% PFA at 4°C. Kidneys were then transferred into 15% Sucrose in PBS and rocked at room temperature for 30 mins followed by rocking overnight at 4˚C in 30% sucrose. Next, kidneys were placed into a 1:1 mixture of 30% sucrose/PBS and OCT and rocked at 4˚C for 2 hours and then were embedded in OCT and stored at -80˚C until sectioned. Cryosections (7uM) were obtained using a Leica Cryostat. Nuclei were counterstained with VECTASHIELD Antifade Mounting Medium with DAPI (Vector Laboratories, Burlingame, CA, USA). Images were obtained with a laser scanning confocal microscope (LSM780) and the ZEN2010 software (Carl Zeiss Canada Ltd., Toronto, ON, Canada) at room temperature and processed by Adobe Photoshop and Illustrator software.

### Fluorescence activated cell sorting (FACS)

Whole embryonic kidneys were isolated and activated with tamoxifen as previously described. Cells were then washed in PBS and re-suspended into 500 μL of 2% FBS in PBS solution and kept at 4˚C until they were sorted. Cell sorting was performed by immunophenotyping core facility staff using a BD FACSAria Fusion. Isolated Cited1/Tom cells isolated were immediately pelleted and frozen at -80˚C.

### Droplet digital PCR (ddPCR)

RNA was extracted from Cited1/Tom cells followed by cDNA synthesis as previously described (n = 4). Droplets were formed in a QX200 Droplet Generator and PCR was performed using the QX200 ddPCR EvaGreen Supermix (Bio-Rad, Mississauga, ON, Canada) and specific primer sets in a C1000 Touch Thermal Cycler (Bio-Rad, Mississauga, ON, Canada). Droplets were read using the QX200 Droplet Reader machine and results were displayed in QuantaSoft software.

### Statistical analysis

Graphs are presented as mean ± SEM of three or more independent results. Statistical significance was assessed by a one-way ANOVA followed by a Dunnett correction for multiple comparisons. ddPCR results were analyzed by unpaired t-tests.

## Results

### M15 cells

**Fig 1:**
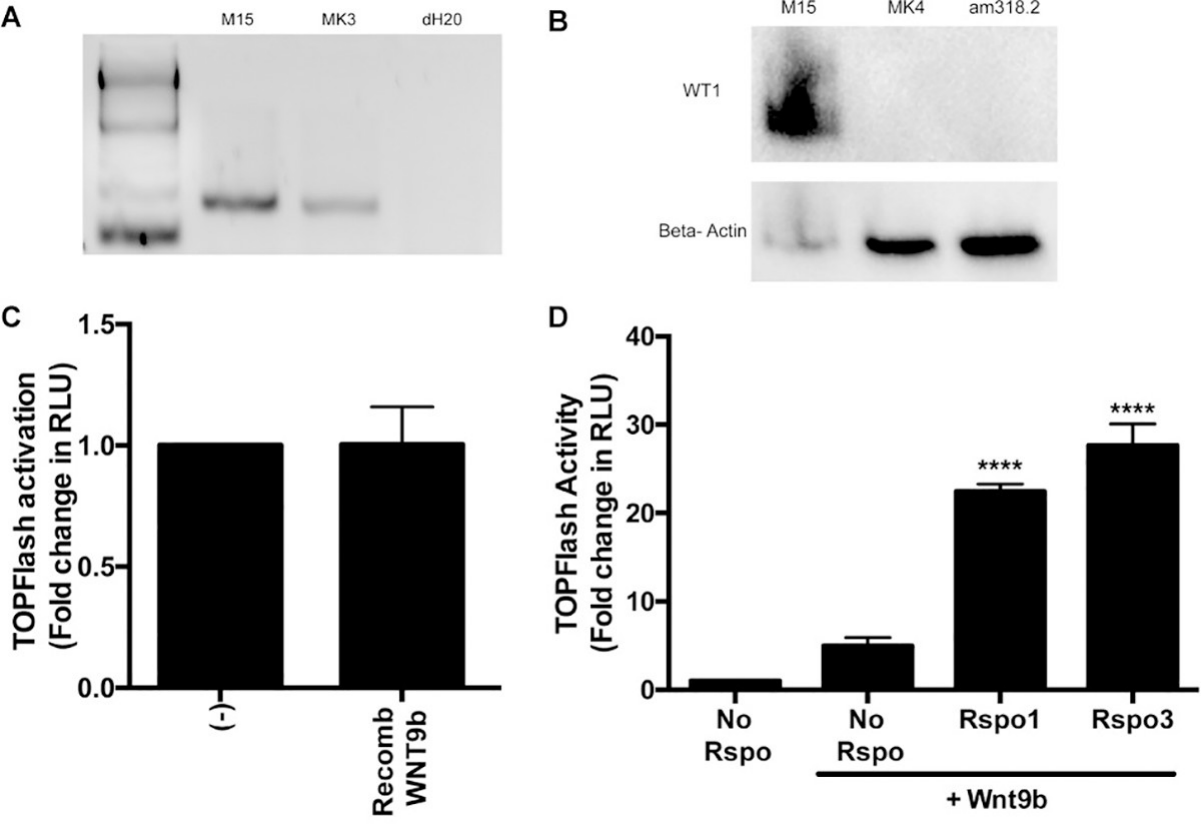
Effect of recombinant RSPO1 on responsiveness of M15 cells to WNT9b. (A) mRNA from E10.5 mouse mesonephric mesenchyme (M15 cells) was analyzed by RT-PCR for *Wt1* mRNA expression in M15 cells and MK3 (positive control) cells vs water blank. (B) Lysates of M15 cells vs E14.5 MK4 (negative control) or am318.2 mesenchymal stem cells from 20-week gestation human amniotic fluid were analyzed by Western immunoblotting for WT1 protein (upper panel) and Beta actin (lower panel). (C) M15 cells were transiently transfected with β-catenin-luciferase reporter (TOPFlash) and Renilla-luciferase reporter. The cells were exposed to recombinant WNT9b (50 ng/ml). After 48 hours, TOPFlash to Renilla signal (RLU) was measured in a luminometer. An unpaired two-tailed Welch’s t-test was performed. (ns) p = 0.98. (D) M15 cells were transfected with TOPFlash, Renilla and *Wnt9b* plasmids and cultured for 24 hours; recombinant RSPO1 or RSPO3 (200 ng/ml) were added for an additional 24 hours and TOPFlash to Renilla signal was measured. A one-way ANOVA followed by a Dunnett correction for multiple comparisons was performed. (****) = p <0.0001.

**A** committed lineage of NPCs emerge from the OSR1(+)/WT1(+) intermediate mesoderm as early as embryonic day E7.5 [1]. To model early events that render NPCs responsive to the inductive WNT9b signal from ureteric bud, we analyzed the M15 cell line. M15 cells are derived from E10.5 mesonephric mesenchyme of mice bearing the large T protein polyoma virus under the control of an early viral enhancer [17]. These cells are thought to represent the NPC phenotype one day prior to arrival of the ureteric bud at E11.5. To validate the lineage specification of M15 cells, we confirmed the expression WT1 (Fig 1A and 1B) and the pattern of additional transcripts characteristic of the early NPC lineage (Table 1). We detected transcripts of key early NPC markers including *Osr1* and *Cited1* but not markers of NPCs after exposure to the ureteric bud, such as *Wnt4* and *Rara* (Table 1). We then screened M15 cells for mRNA expression (RT-PCR) of candidate genes in the canonical WNT/β-catenin signaling pathway (Table 1). We identified expression of β-Catenin, *Lrp*6, *Lgr4/6* and *Fzd1-6*. Notably absent were *Rspo1* and 3, *Fzd7*-*10*, *Lrp5* and *Lgr5*.

**Table 1:** mRNA expression of WNT/β-catenin pathway components in M15 cells.

Wnt-signalling genes				NPC markers	
Gene	Expression	Gene	Expression	Gene	Expression
*Fzd1*	+	*Lrp5*	-	*WT1*	+
*Fzd2*	+	*Lrp6*	+	*Osr1*	+
*Fzd3*	+	*Rspo1*	-	*Cited1*	+
*Fzd4*	+	*Rspo3*	-	*Six2*	-
*Fzd5*	+	*β-catenin*	+	*Wnt4*	-
*Fzd6*	+	*Wnt9b*	-	*Rara*	-
*Fzd7*	-	*Lgr4*	+		
*Fzd8*	-	*Lgr5*	-		
*Fzd9*	-	*Lgr6*	+		
*Fzd10*	-				

### M15 cells are unresponsive to external WNT9b

To ascertain whether M15 cells are primed to respond to a WNT9b signal, we transiently transfected the cells with TOPFlash, a β-Catenin/luciferase reporter, and exposed them to recombinant WNT9B protein at concentrations ranging from 50–400 ng/ml but detected only minimal response (1.05-fold) (Fig 1C).

### RSPO1 enhances responsiveness of M15 cells to WNT9b

Considering M15 cells lack both R-spondins known to be expressed in NPCs of embryonic mouse kidney cap mesenchyme (GUDMAP), we reasoned that M15 cell WNT-responsiveness might be limited by the stability of the WNT-receptor complex at the cell surface [23–25]. To test this hypothesis, we first transfected M15 cells with TOPFlash and assessed the response to a co-transfected WNT9b expression plasmid. As seen in Fig 1D we detected a significant (5-fold) increase in luciferase activity. We then added recombinant RSPO1 (200 ng/ml) or RSPO3 (200 ng/ml) which further increased the signal to 22- and 27-fold above baseline, respectively (p<0.0001) (Fig 1D). Preliminary dose-response studies showed that no further signal increase was obtained with higher concentrations of either R-spondin protein. To dissect the importance of other canonical WNT-pathway components, we added *Wnt9b* plasmid and recombinant RSPO1 (200 ng/ml) in all subsequent experiments.

### Frizzled receptor expression in cap mesenchyme

**Fig 2:**
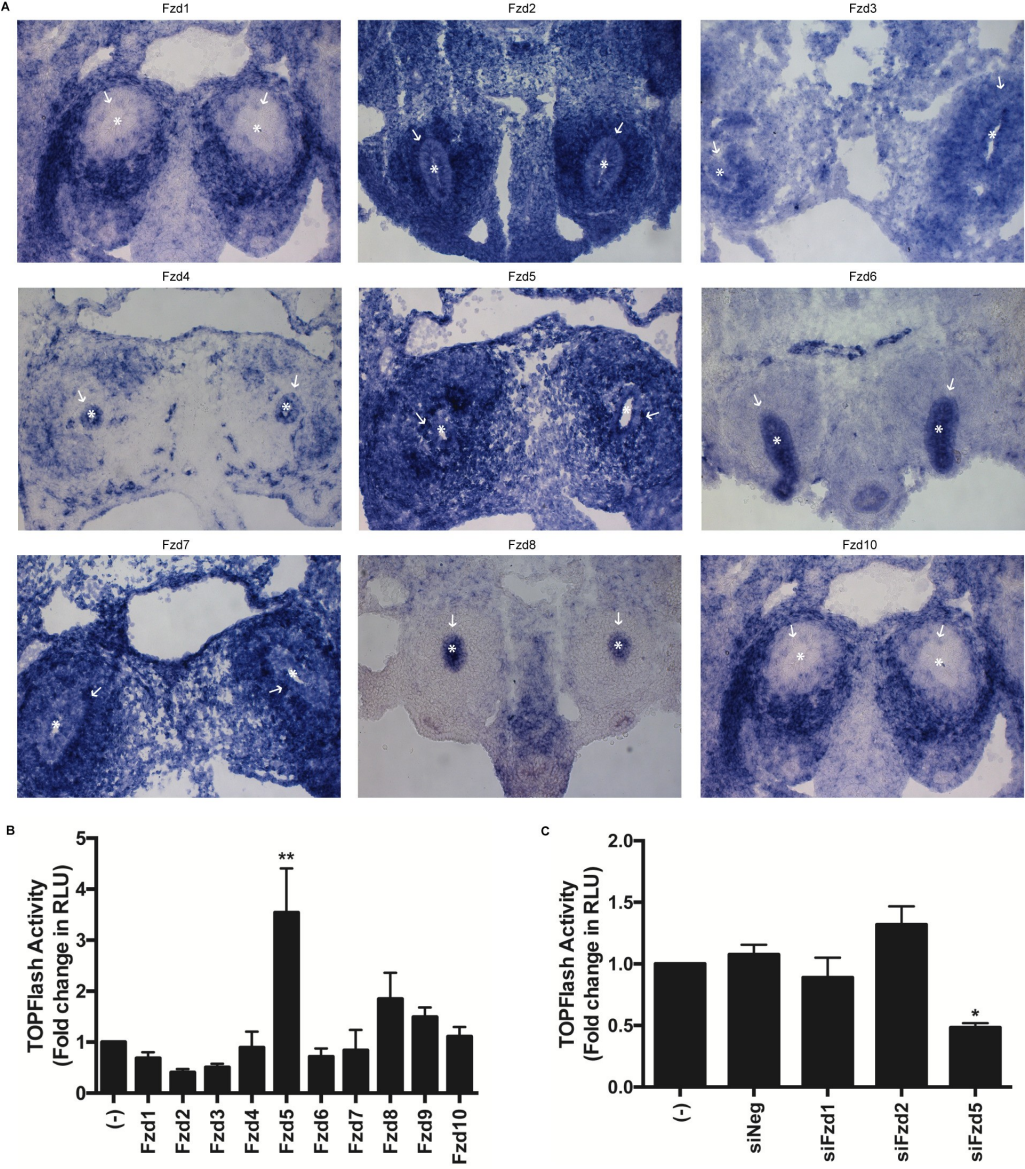
Effect of *Fzd* expression on WNT9b responsiveness in M15 cells. (A) Cross sections of E11.5 embryos displaying both nephric fields were assessed by *in situ* hybridization using riboprobes for *Fzd1-10*, except *Fzd9* which was unsuccessful for technical reasons. Asterisk (*) marks ureteric buds. Arrow (→) marks cells of the cap mesenchyme. (B) M15 cells were transiently transfected with β-catenin-luciferase reporter (TOPFlash), Renilla-luciferase reporter, *Wnt9b*-expression vector and various *Fzd*1-10 expression plasmids in the presence of recombinant RSPO1 (200 ng/ml). TOPFlash to Renilla signal was measured after 48 hours. A one-way ANOVA followed by a Dunnett correction for multiple comparisons was performed. (**) p = 0.0002 (C) M15 cells were transiently transfected with β-catenin-luciferase reporter (TOPFlash), Renilla-luciferase reporter, *Wnt9b*-expression vector and siRNAs targeting *Fzd*1, *Fzd*2 or *Fzd*5 vs a scrambled negative control siRNA in the presence of recombinant RSPO1 (200 ng/ml). TOPFlash to Renilla signal was measured. A one-way ANOVA followed by a Dunnett correction for multiple comparisons was performed. (*) p = 0.005.

To identify candidate Frizzled receptors responsible for transducing the WNT9b response in NPCs, we performed *in situ* hybridization for the Frizzled family members (*Fzd1-10)* in E11.5 mouse kidney, except for *Fzd9* which was unsuccessful. As seen in Fig 2A, embryos cross-sectioned across both nephric fields show several Frizzled family members (*Fzd2*, *Fzd3*, *Fzd5 and Fzd7*) with diffuse expression patterns but with concentrated expression in the cap mesenchyme; in contrast to *Fzds* with weak expression in the cap mesenchyme (*Fzd4* and *Fzd10*) or strong expression restricted to ureteric bud branch tips (*Fzd6* and *Fzd8*).

### Transfection of M15 cells with *Fzd5* enhances WNT9b responsiveness

To confirm whether one of the *Fzd* receptors is rate limiting in M15 cells, we transfected each member of the *Fzd* receptor family (*Fzds 1–10*) individually into M15 cells expressing TOPFlash. Cells were co-transfected with *Wnt9b* and exposed to recombinant RSPO1 (200 ng/ml) in each experiment. As seen in Fig 2B, the only *Fzd* which significantly augmented WNT9b-induced TOPFlash signal was *Fzd5*. When M15 cells were co-transfected with *Fzd5*, activity of the canonical WNT/β-Catenin reporter was increased 3.5-fold (p = 0.0002). We then performed similar experiments in M15 cells co-transfected with an siRNA targeting *Fzd5*, previously shown to knock down *Fzd5* expression level by 70%. As seen in Fig 2C, presence of the *Fzd5* siRNA reduced WNT9b-dependent TOPFlash activity by 52% (p = 0.005), whereas knockdown of *Fzd1* and *Fzd2* resulted in non-significant changes.

### *Lrp6* is required for optimal responsiveness of M15 cells to WNT9b

**Fig 3:**
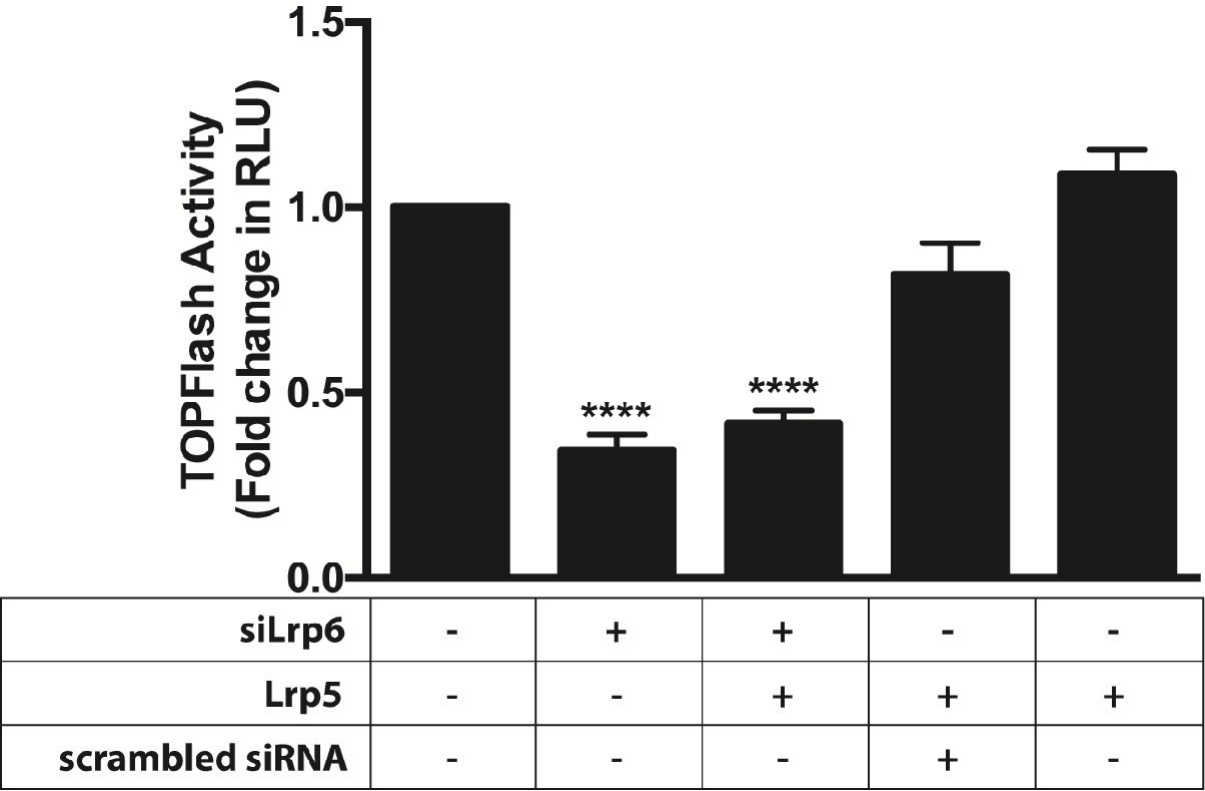
*Lrp6* is required for optimal responsiveness of M15 cells to WNT9b. M15 cells were transiently transfected with β-catenin-luciferase reporter (TOPFlash), Renilla-luciferase reporter and a *Wnt9b* expression vector and treated with RSPO1 (200 ng/ml). The cells were co-transfected with an siRNA targeting *Lrp*6 or a scrambled negative control siRNA in the presence of recombinant RSPO1 (200 ng/ml). After 48 hours, TOPFlash to Renilla signal was measured. In another experiment, the cells were co-transfected with a *Lrp5* expression plasmid to assess its effect on WNT9b pathway activity. A one-way ANOVA followed by a Dunnett correction for multiple comparisons was performed. (****) p<0.0001.

To examine the importance of *Lrp* expression to the canonical WNT9b-responsiveness, we transiently transfected M15 cells with *Wnt9b*, TOPFlash and a *Lrp6* siRNA. A scrambled siRNA was transfected in another condition as a control. As seen in Fig 3, addition of the *Lrp6* siRNA reduced WNT9b-dependent TOPFlash signal by 66% (p<0.0001) whereas the scrambled siRNA had no effect. Interestingly, additional co-transfection with *Lrp5* was unable to rescue WNT9b pathway activity in the presence of *Lrp6* siRNA. Co-transfection of M15 cells with *Lrp5* (in the absence of siRNA) had no effect on its own.

### Responsiveness to extrinsic WNT9b is restored by addition of *Fzd5* and RSPO1

**Fig 4:**
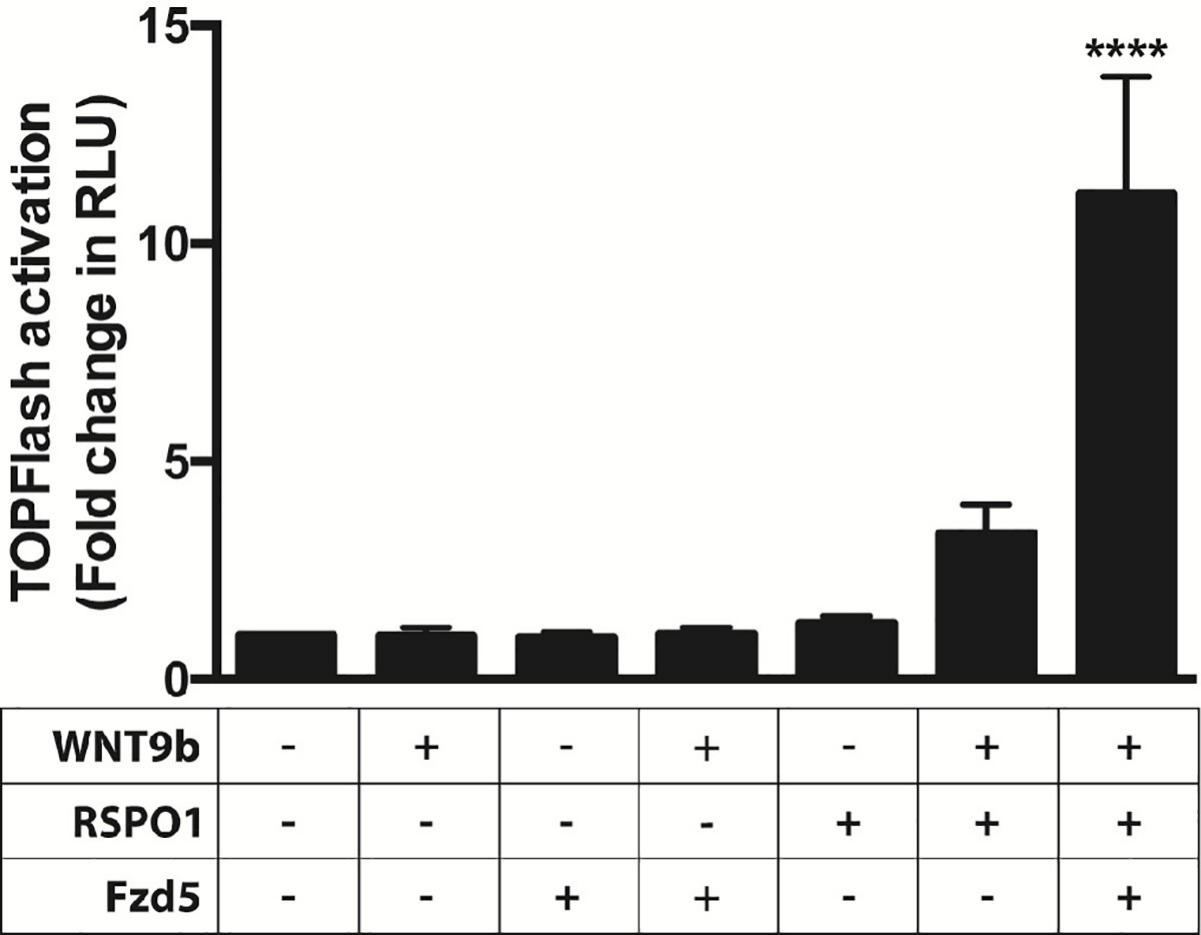
Responsiveness to extrinsic WNT9b is restored by addition of *Fzd* and RSPO1. In all conditions, M15 cells were transiently transfected with β-catenin-luciferase reporter (TOPFlash) and Renilla-luciferase reporter; in some experiments the cells were co-transfected with *Fzd*5 expression plasmid. TOPFlash to Renilla signal was measured in the presence or absence of recombinant WNT9b (50 ng/mL) and/or recombinant RSPO1 (200 ng/ml). A one-way ANOVA followed by a Dunnett correction for multiple comparisons was performed. (****) = p<0.0001.

To ascertain whether M15 cell responsiveness to an external source of WNT9b could be restored by addition of suboptimal WNT-pathway components, we transfected the cells with TOPFlash and *Fzd5*. We then treated them with recombinant RSPO1 and measured luciferase activity. As seen in Fig 4, no response was detected in cells exposed to WNT9b, RSPO1 or *Fzd5* alone. However, the signal was increased 3.3-fold over baseline in M15 cells exposed to recombinant WNT9b and RSPO1. The signal was increased to 11.1-fold over baseline in M15 cells transfected with *Fzd5* and exposed to recombinant WNT9b and RSPO1 (p<0.0001) (Fig 4).

### Cited1 cells isolated from embryonic mouse kidney express *Wt1*, *Fzd5*, *Lrp6* and *Rspo1*

**Fig 5:**
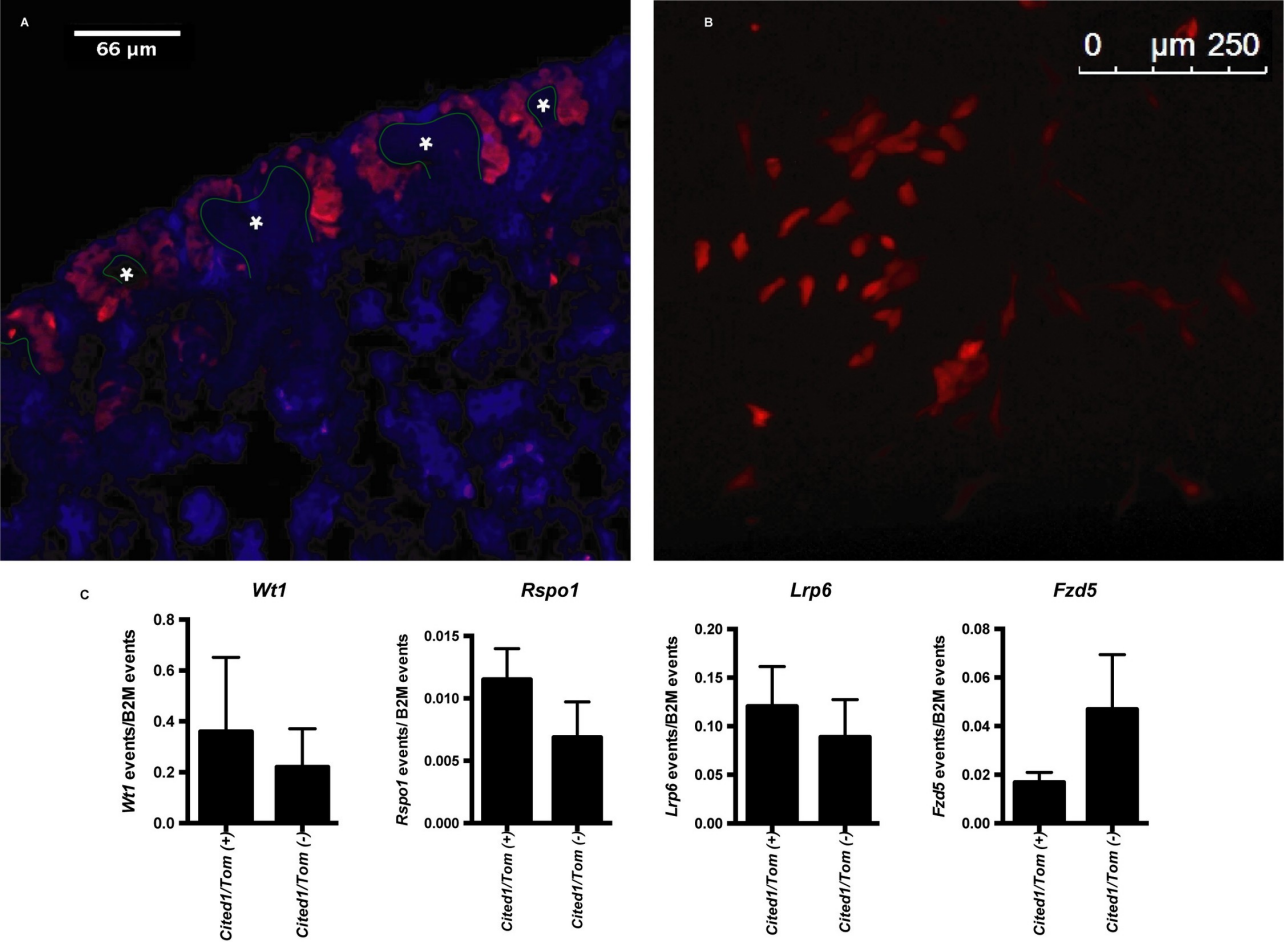
Identification and isolation of Cited1 expressing cells from embryonic kidneys. (A) Cryosections of E18 embryonic kidneys isolated from Cited1^Cre^/TomatoRed mice were assessed by immunofluorescent microscopy for the presence of TomatoRed in cap mesenchyme surrounding ureteric bud tips. (*) Ureteric Bud outlined in green. (B) Whole E15.5 embryonic kidneys were dispersed into monolayer culture in the presence of tamoxifen (2.5 μg/ml) for 16 hours and TomatoRed(+) cells were visualized by immunofluorescent microscopy. **(**C) Expression of WNT9b pathway component transcripts in Cited1^Cre^/TomatoRed cells isolated by FACS from E15.5 embryonic mouse kidney quantified by ddPCR. Bars represent mean number of events of gene of interest normalized to B2M events (n = 2, 17 total pooled embryonic kidneys). Error bars represent standard error of the mean.

To confirm expression of the key components of the WNT9b signaling pathway identified above in a primary NPC, we isolated Cited1-expressing cells from embryonic mouse kidneys. Six2 is a commonly used cap mesenchyme marker, however, Cited1 has been shown to have overlapping expression with Six2 and also is downregulated before NPCs begin differentiation into mature tubules [26, 27]. To identify Cited1 cells in the cap mesenchyme, we crossed mice with a floxed tdTomato (TomatoRed) transgene to mice bearing a tamoxifen-inducible *Cited1*-driven Cre Recombinase [20]. The Cited1-Cre mouse also contains EGFP, however, we were not able to specifically isolate the Cited1 population of cells due to high green autofluorescence observed in the kidney. As seen in Fig 5A, tamoxifen administered to the pregnant mother at E17 activated TomatoRed in NPCs of the cap mesenchyme. Although activation of the Cre-recombinase was successful in vivo, this method required more time between tamoxifen injection and cell isolation which increased the likelihood of including differentiated cells into our analysis. To circumvent this issue and isolate NPCs rapidly after activation of the TomatoRed tag, we digested E15.5 embryonic kidneys from *Cited1*^*Cre*^/TomatoRed mice with collagenase, dispersed the cells into monolayer culture and added 4-hydorxytamoxifen (2.5μg/ml) to induce Cre-recombinase expression *in vitro* (Fig 5B). After 12 hours, TomatoRed(+) cells were isolated by FACS for analysis. This method ensured that fewer red-labelled cells would differentiate before FACS isolation. We extracted RNA from Cited1/TomatoRed(+) cells of 17 embryonic kidneys pooled from 4 litters (two litters per sample; sample 1: n = 9 embryonic kidneys; sample 2: n = 8 embryonic kidneys) and analyzed transcripts levels by droplet digital PCR (ddPCR) due to the limited number of cells isolated per kidney. As seen in Fig 5C, we confirmed mRNA expression of *Wt1*, *Fzd5*, *Rspo1* and *Lrp6* in the *Cited1*/TomatoRed(+) NPCs from E15.5 cap mesenchyme. Each condition was compared to the Cited1/TomatoRed(-) fraction of cells obtained from the same kidneys and normalized to beta-2-microglobulin (B2M) transcript levels. As the Cited1/TomatoRed(+) population of cells represents approximately 6% of the E15.5 kidneys after FACS, we expected the Cited1/TomatoRed(-) population of cells to also express some level of our markers of interest, therefore we used this condition as a positive control.

## Discussion

Around embryonic day E9.0 of mouse development, a lineage of WT1-expressing progenitor cells emerge within the OSR1(+) intermediate mesoderm. To model this early NPC prior to the arrival of the ureteric bud, we studied the M15 cell line isolated from E10.5 mouse kidneys [17]. These cells express *Osr1*, WT1 and *Cited1*, placing them in the early NPC lineage. Previous studies from our lab showed the essential role of WT1 for responsiveness to the inductive WNT9b signal through suppression of EZH2, a histone H3K27 methyltransferase. EZH2 suppression in turn opens up chromatin, permitting exit from the stem cell state [15, 28]. Thus, WT1 is essential for maturation of the nephron progenitor cell lineage. Nevertheless, we found that M15 cells were unresponsive to WNT9b *in vitro*. This suggests that WT1 expression alone is not sufficient to prime the NPC for WNT-responsiveness and that the early NPC must acquire additional molecular properties by the time the ureteric bud arrives at E10.5-E11.

Although M15 cells are unresponsive to WNT9b, they are derived from the Osr1/WT1(+) lineage in embryonic kidney and afford an informative *in vitro* model in which to explore the molecular basis for WNT9b responsiveness. M15 cells express many components of the canonical WNT-signaling pathway, including 4 frizzled receptors (*Fzd1*, *Fzd2*, *Fzd3* and *Fzd5*) which can be detected in the cap mesenchyme surrounding each ureteric bud tip. M15 cells also express the frizzled co-receptor *Lrp6* and complex-stabilizing proteins *Lgr4/6*, shown by the GUDMAP consortium to be present in cap mesenchyme [24, 25]. Strikingly, however, they do not express members of the R-spondin family. Several investigators have shown that canonical WNT-signal transduction is dramatically increased by stabilization of the FZD/LRP6/WNT complex at the cell surface as a result of the presence of R-spondins [23].

The R-spondin family binds to the WNT-receptor complex through its association with an LGR family member [29] and ZNRF3/RNF43. ZNRF3 is a negative regulator of canonical WNT-signalling and has a role of ubiquitinating FZD receptors, targeting them for destruction and also preventing phosphorylation of LRP receptors, keeping them in their inactive form [30, 31]. RSPO1 binds to ZNRF3 which in turn associates with an LGR receptor to remove ZNRF3 from the cell membrane and allows the WNT-receptor to remain active at the cell surface [31]. RSPO1 transcripts are strongly expressed in the cap mesenchyme of E11.5 mouse kidney [32] but were entirely absent in M15 cells. In our study, pre-treatment of M15 cells with RSPO1 enhanced WNT9b-induced canonical signaling activity 4-fold. When the cells were transfected with additional *Fzd5*, RSPO1 augmented WNT9b-responsiveness 11-fold. Thus, RSPO1 appears to be critical for a robust response to WNT9b and its absence in M15 cells precludes measurable signal transduction. We postulate that RSPO1 is not expressed in the early developing kidney (E10.5) and the effects of WT1 on NPC chromatin alone are insufficient to induce RSPO1 expression. RSPO1 expression may be a late priming event in the maturation of the NPC.

The effects of RSPO1/LGR interactions are crucial for normal nephrogenesis. Three LGRs (*Lgr4*, *Lgr5*, *Lgr6*) interact with R-spondin proteins [29, 33–36]. LGR5 has been well-studied in intestinal epithelia where it was shown to have an important function to promote intestinal stem cell renewal [37–39]. We detected both Lgr4 and Lgr6 transcripts in M15 cells which is in concordance with the data found on GUDMAP, where expression was detected in the NPC lineage. Current commercially available siRNAs are non-specific and result in knockdown of both transcripts, therefore we cannot determine which protein is most important in NPCs. Another group studying *Lgr4*-knockout mice observed increased apoptosis in NPCs and disruption of the process by which NPCs condense around ureteric bud tips [40], suggesting Lgr4 may be the primary determinant of WNT9b signal transduction in cap mesenchyme. In contrast, murine knockout of the *Rspo1* gene has no renal phenotype [41], likely reflecting redundancy between RSPO1 and RSPO3, both of which are expressed in the cap mesenchyme (GUDMAP). This is in keeping with our *in vitro* observations indicating that both recombinant RSPO1 and RSPO3 enhance WNT-responsiveness in M15 cells.

Few studies have investigated frizzled expression in the developing kidney. Ureteric bud specific expression of FZD4 and FZD8 in E11.5 kidneys was previously examined using *Fzd4*-lacZ and *Fzd8*-lacZ mouse models [42]. Additionally, widespread renal expression of FZD2 and FZD7 was observed in 12, 13 and 18-week human fetal kidneys [43]. Our in situ hybridization data revealed distinct Frizzled expression patterns in E11.5 mouse kidneys. We detected *Fzd1*, *Fzd2*, *Fzd3*, *Fzd5* and *Fzd7* expression in the cap mesenchyme, whereas *Fzd4*, *Fzd6* and *Fzd8* expression was highly restricted to the ureteric bud. *Fzd10* expression was relatively non-specific and *Fzd9* in situ hybridization did not work for technical reasons. Interestingly, we found that in the presence of RSPO1, only *Fzd5* was limiting the WNT-response as the canonical signal was amplified by transfecting cells with *Fzd5* but none of the other Fzd family members. Furthermore, siRNA knockdown of *Fzd5* (but not *Fzd1* or *Fzd2*) reduced WNT9b responsiveness. These observations suggest that FZD5 is the primary WNT co-receptor involved in transducing the inductive WNT9b signal in mammalian kidney. Moreover, it raises the possibility that the other FZDs expressed in cap mesenchyme might be involved in transduction of other canonical and non-canonical WNT ligands, such as WNT6 and WNT11 from ureteric bud tips [44, 45] or WNT2b and WNT4 from the metanephric mesenchyme of the developing kidney [46, 47].

Phylogenetic analysis of human frizzled proteins established five distinct frizzled subgroups [48], one of which consisted of FZD5 and FZD8. Our in situ hybridization studies of E11.5 embryonic mouse kidney demonstrate expression of *Fzd5* in the cap mesenchyme while *Fzd8* is exclusively expressed in the ureteric bud. Interestingly, WNT9b was demonstrated to bind and form a complex with FZD8 and LRP6 [49]. It is conceivable that *Fzd8* mediates the robust canonical WNT signaling activity in ureteric buds reported by Bridgewater and Iglesias [13, 50].

The renal stroma, marked by the *Foxd1* promoter, is another major compartment of the developing kidney which surrounds NPCs in the cap mesenchyme. *Foxd1* knockout mice develop smaller kidneys with disorganized tubular structures suggesting the Foxd1(+) stroma is required for nephrogenesis to proceed normally [51–53]. Das et al (2013) propose a model in which the renal stroma promotes NPC differentiation through secretion of Fat4. Ultimately, this process results in phosphorylation of YAP/TAZ which promotes transcription of Class I beta-catenin targets (differentiation) rather than Class II beta-catenin targets (self-renewal) [51]. In Foxd1 knockout mice, NPCs do not receive the Fat4 signal from the renal stroma and remain in a state of self-renewal. However, initiation of differentiation or self-renewal both require Wnt9b to bind to its cell surface receptor.

**Fig 6:**
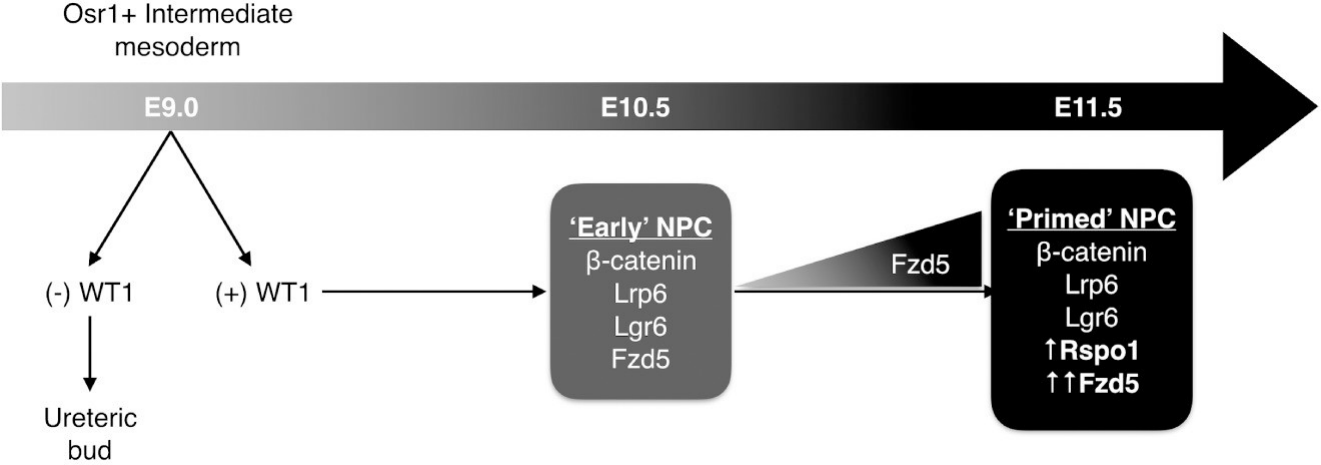
Proposed model of nephron progenitor cell development in embryonic mouse kidney. Early WT1(+) NPCs express a number of important molecules in the canonical WNT-signalling pathway. By E11.5, increased expression of Fzd5 and addition of Rspo1 render NPCs fully competent to respond to the inductive WNT9b signal.

Based on our data and the observations above, we propose a model of renal development in which WT1(+) NPCs in E10.5 embryonic mouse kidney express some, but not all, components of the canonical WNT-signaling pathway. By E11.5, additional events (expression of RSPO1 and increased expression of FZD5) have primed NPCs forming the cap mesenchyme, allowing responsiveness to the anticipated WNT9b signal from ureteric bud (Fig 6).

## Supporting information

S1 File ARRIVE guidelines checklist. (PDF)
